# Knowledge, Attitude, and Practices Among Healthcare Practitioners in the Context of Multidrug Resistance Tuberculosis: An Appraisal to Disease Elimination

**DOI:** 10.7759/cureus.36788

**Published:** 2023-03-28

**Authors:** Mantu Jain, Sutapa Rath, Monalisa Mohanty, Baijayantimala Mishra, Prasanta R Mohapatra

**Affiliations:** 1 Orthopedics, All India Institute of Medical Sciences, Bhubaneswar, IND; 2 Microbiology, All India Institute of Medical Sciences, Bhubaneswar, IND; 3 Pulmonary Medicine and Critical Care, All India Institute of Medical Sciences, Bhubaneswar, IND

**Keywords:** healthcare practitioner, national tuberculosis elimination program, genexpert assay, antitubercular therapy, multidrug-resistant tuberculosis

## Abstract

Introduction: Tuberculosis (TB) remains one of the leading infectious causes of death worldwide, and India is among the countries with the highest TB burden. TB control is facing several roadblocks in our country with the rapid development of multidrug-resistant (MDR) as well as extensively drug-resistant TB (XDR) and as an after-effect of the global COVID-19 pandemic. With the target of TB elimination by 2025 (National Tuberculosis Elimination Program, NTEP), there is a need that treating physicians in our country be well aware of MDR-TB and be able to diagnose and treat it at an appropriate time. The present study is conducted to explore the knowledge levels, attitudes, and practices concerning MDR-TB amongst healthcare professionals working in different healthcare sectors.

Methods: A total of 250 allopathic medical practitioners (Bachelor of Medicine and Bachelor of Surgery [MBBS], specialists, and superspecialists) working in any sector (private or government), who are directly involved in managing any form of TB patient and are willing to undertake the assessment, were included in this online questionnaire-based survey that was circulated using various social media platforms like WhatsApp, Facebook, Linked In, and Gmail. Responses to the questionnaires created in Google Forms were analyzed by capturing data in a Microsoft Excel® spreadsheet for further statistical analysis. The data were analyzed using multiple measures of dispersion and cross-tabulations.

Results: Among the 250 participants, most of the participants had encountered MDR-TB in their clinical practice, and the majority believe that MDR-TB is a rising problem. Although 88% of the participants did a GeneXpert assay before the start of anti-tubercular therapy (ATT), three-fourths of the participants knew that the assay detects the MTB genome and rifampicin resistance. MDR-TB was suspected in participants after no clinical improvement was observed after 3-6 weeks of a trial of ATT. Two-thirds of the participants knew that linezolid is currently being used as a second-line drug for the treatment of MDR- TB. The respondents in our survey mostly do not themselves treat MDR-TB and refer the patients to an MDR-TB center or a pulmonary medicine specialist.

Conclusion: Healthcare practitioners (HCPs) with good knowledge levels can diagnose and treat TB patients appropriately, thus decreasing the rising MDR-TB problem, and they can educate patients and the general population about TB and the emerging MDR-TB situation. With the current level of knowledge about MDR-TB management, there is certainly an urgent need for educational and persuasive measures for the training of doctors in both the public and private sectors so as to achieve TB elimination by 2025.

## Introduction

Despite the advancement of diagnostic medical technology, tuberculosis (TB) caused by the bacilli *Mycobacterium tuberculosis*, remains one of the leading infectious causes of death worldwide. In 2020, an estimated 9.9 million cases and 1.5 million deaths were attributed to TB. India bears the highest TB burden in the world, contributing nearly 27% of TB cases [1]. Even with a robust national TB elimination program (NTEP) in place and the availability of antitubercular therapy (ATT), controlling TB in our country is a huge challenge with the development of multidrug-resistant (MDR) as well as extensively drug-resistant TB (XDR). As per the India TB Report - 2022, the estimated number of MDR and XDR-TB in India was four per 100,000 and one per 100,000 population, respectively [2]. With the mission of eliminating TB by 2025 (NTEP) [3] and these dismaying figures for MDR-TB, there is a need that treating clinicians in our country be well aware of MDR-TB and be able to diagnose and treat it at an appropriate time to prevent further transmission.

It is usually presumed that clinicians, being frontline healthcare practitioners (HCPs), have the basic knowledge regarding MDR-TB and its sequelae. However, studies suggest existing knowledge gaps regarding the suspicion, diagnosis, and treatment of MDR and XDR-TB [4,5]. This present study is conducted to explore the knowledge levels, attitudes and practices concerning MDR-TB amongst clinicians working in different health care sectors. Secondly, the findings from this study could be used by decision-makers and institutional managers to design and implement interventions to address the shortcomings that will be identified.

## Materials and methods

Study design

The study was conducted after obtaining institutional ethics (Institutional Ethics Committee, All India Institute of Medical Sciences, Bhubaneswar) approval (letter no- T/IM-NF/ Micro/22/16). It was an online questionnaire-based survey (https://docs.google.com/forms/d/e/1FAIpQLSf7pn96FrkmtUM9iQKNmjZbN0gPPjKjYXbS7wB8-VFw77E_w/viewform?vc=0&c=0&w=1&flr=0 ) and sent to all medical residents, and practitioners using various social media platforms like WhatsApp, Facebook, Linked In, and Gmail. The questionnaire contained a total of 20 questions which were either short answer or multiple-choice questions and prepared on Google Forms. The questionnaire was finalized by three experts after multiple rounds of brainstorming sessions and taking into consideration existing published materials, data from the Index TB guidelines (ITBG) and the World Health Organization (WHO) MDR-TB management guidelines [1-8]. Knowledge about MDR-TB was assessed by asking questions regarding MDR-TB, symptoms, diagnosis, and treatment modalities.

Study population

Only allopathic medical practitioners (Bachelor of Medicine and Bachelor of Surgery (MBBS), specialists and superspecialists) working in any sector (private or government), who are directly involved in managing any form of TB patients and were willing to undertake the assessment were included in this study.

Exclusion Criteria

Allopathic doctors who did not give complete information and failed to answer all questions and nonallopathic medical practitioners including Ayurveda, Yoga and Naturopathy, Unani, Siddha and Homeopathy (AYUSH) practitioners were excluded from the study.

Sample size

The sample size was calculated using open-epi software. Considering the error margin of 10% and the response rate of 20%, the questionnaire was distributed to a minimum of 250 prospective participants.

Data analysis

The responses to the questionnaires created in the Google Forms were analyzed for all the participants. The data were captured in a Microsoft Excel® spreadsheet for further statistical analysis. Data were analyzed using multiple measures of dispersion and cross-tabulations. Categorical data were presented as percentages or frequencies as appropriate. Subgroup analysis was done to compare various specialties for better problem identification. A cross-tabulation was performed in order to assess the association between variables. The level of statistical significance was set at ≤ 0.05.

## Results

The study was completed in two months (August, 2022-September, 2022). A total of 250 doctors from all over the country participated in our study.

Participants characteristics

Most of the participants (44.4%) belonged to the 31-40 years age group. About 66% of the respondents were male. The majority of respondents were specialists (71.2%), with few general practitioners (14 %) and super-specialists (14.8%). The distribution of participants in terms of experience was almost similar between the categories; less than five years of practice (27.2%); 5-10 years (35.6%) and >10 years (37.2%) of practice. The majority of the participants belonged to the orthopedic department (28.4%) followed by the pulmonary department (17.6%) and the medicine department (12.4%) and 65.2% of the participants belonged to a government set-up (Table 1).

**Table 1:** Socio-demographic characteristics of healthcare providers included in the study

	Items	class	Number	Percentage
1	Age group in years	<30	37	14.8
		31-40	111	44.4
		41-50	64	25.6
		>50 y	38	15.2
2	Sex	Male	164	65.6
		Female	86	34.4
3	Qualification	General practioners	35	14
		Specialist	178	71.2
		Super specialist	37	14.8
4	Speciality	Pulmonary/Chest	44	17.6
		Orthopaedics/Spine	71	28.4
		Neurosciences (Neurology/ neurosurgery)	3	1.2
		Urology	5	2
		General surgery	16	6.4
		Gastology/Gastrosurgery	1	0.4
		General medicine ( Medicine Specialists)	31	12.4
		Paediatrics	19	7.6
		Obstretics & Gynecology	15	6
		Microbiology	14	5.6
		ENT	2	0.8
		Fetal medicine	1	0.4
		Spine Surgery	1	0.4
		Community medicine	1	0.4
		Infectious Disease	2	0.8
		Paediatric Surgery	1	0.4
		Radiologist	1	0.4
		Oncosurgery	1	0.4
		Pathology	1	0.4
		Pharmacology	1	0.4
		Psychiatry	2	0.8
		Not applicable	17	6.8
5	Experience in years	<5	68	27.2
		5-10	89	35.6
		>10	93	37.2
6	Workplace	Government tertiary care hospital with medical college	124	49.6
		Government tertiary care hospital without medical college	16	6.4
		Government CHC or PHC	23	9.2
		Private multispecialty hospital	67	26.8
		Independent practice	14	5.6
		Private medical college and super speciality hospital	4	1.6
		Private tertiary super speciality hospital	2	0.8

Knowledge regarding MDR-TB

TB sensitization programs had been attended by 71.6% of the participants in the past (Table 2).

**Table 2:** Knowledge, awareness and attitude of healthcare providers toward multidrug-resistant tuberculosis and extra-pulmonary tuberculosis ITBG: Index TB Guidelines; EPTB: Extrapulmonary tuberculosis

S/L no	Items		Number	Percentage
1	Have you attended any sensitisation program for TB	Yes	179	71.6
		No	71	28.4
2	Are you aware of ITBG for treating EPTB	Yes	225	90
		No	25	10
3	Do you follow ITBG for treating EPTB	Always	144	57.6
		Sometimes	90	36
		Never	16	6.4
4	If yes what percentage of patients show response	100%	56	22.4
		75%	161	64.4
		50%	14	5.6
		<50%	19	7.6
5	If no, what is reason for not following ITBG	Not aware of Index- TB guidelines	14	5.6
		Following some other guidelines like institutional guidelines	24	9.6
		Patients lost to follow up or do not show adequate response	15	6
		WHO guidelines are more updated & comprehensive	1	0.4
		Usually, patient directly consulted by the Pulmonologist or Medicine specialist	3	1.2
		Mainly follow NTEP and PMDT	1	0.4
		Not a clinician	4	1.6
		Most of the times I follow, rest of the time based on my experience	2	0.8
		I follow the Index- TB guidelines	186	74.4
6	If a diagnosed case of MDR comes to you, who treats the patients	Pulmonary medicine specialist	87	34.8
		Medicine specialist	8	3.2
		Concerned department	30	12
		Refer to centre for MDR	125	50
7	When do you stop the treatment after recommendation duration	Clinical improvement	21	8.4
		Clinical and radiological improvement	110	44
		Clinical radiological and bacteriological improvement	119	47.6
8	Which MDR-TB you have come across in practice	Lymph node TB/TB Lymphadenitis	146	58.4
		Bone TB/Spinal TB	130	52
		Pleural TB (empyema)	95	38
		CNS TB	78	31.2
		GI TB	64	25.6
		Genitourinary TB	11	4.4
		Tuberculous pericarditis	2	0.8
		Perineal fistula	2	0.8
		Skin	1	0.4
		Not encountered/ Difficult to diagnose	4	1.6
9	When do you suspect MDR-TB during treatment	Radiological increase in lesion	164	65.6
		No clinical improvement after trial ATT (with standard drugs) 3-6 months	216	86.4
		The appearance of draining sinuses, implant failures, etc	100	40
		From the beginning, you start suspecting MDR	50	20
		Sputum positivity even after completion of treatment	142	56.8

These programs have been attended significantly more frequently among the HCPs with experience ≥ 5 years (p-value: 0.0235) (Table 3).

**Table 3:** Comparison of knowledge levels of healthcare practitioners based on levels of experience

Variables		<5 year of Experience (n=68)		≥ 5 year of Experience (n=182)	Odds ratio (95% CI)	P-value
Attended any sensitization programme for Tuberculosis management	Yes	41		138	5.133	0.0235
	No	27		44		
Aware of ITBG for EPTB	Yes	60	165		0.110	0.7402
	No	8	17			
Follow Index-TB guidelines for the treatment of any Tuberculosis	Always + sometimes	60	174		3.342	0.0675
	Never	8	8			
Encounter of MDR TB in practice	Yes	51	151		1.544	0.2140
	No	17	31			
MDR is a rising problem	Yes	64	173		0.088	0.7664
	No	4	9			
Treat MDR TB patients	Yes	34	83		0.228	0.6331
	No	34	99			
Refer MDR TB patients to MDR-Reference center	Yes	33	93		0.048	0.8263
	No	35	89			
Routine Gene Xpert MTB (CBNAAT/ TruNAAT) before starting ATT	Yes	59	163		0.159	0.6903
	No	9	19			
What does Gene Xpert MTB/Rif assay detect	MTB+ Rifampicin Resistance	51	137		0.002	0.9643
	Any other	17	45			
Awareness regarding Use & availability of Second-line drugs	Yes	35	117		2.895	0.0889
	No	33	65			
Awareness of any molecular assay platform available for resistance detection to Second-line drugs	Yes	47	139		1.014	0.3140
	No	21	43			
Awareness of drug sensitivity tests for Anti-TB drugs	Yes	56	162		1.415	0.2343
	No	12	20			
Awareness of use of Linezolid for the treatment of MDR TB	Yes	39	126		2.606	0.1065
	No	29	56			

Yet, only 57.6% of HCPs were religiously following it; and another one-third of HCPs were following it occasionally while the rest were not following the guidelines. Around 80% of the participants had encountered MDR-TB in their clinical practice and the majority (94.8%) believe that MDR-TB is a rising problem. TB lymphadenitis (58.4%), and osteoarticular TB and spinal TB (52%) were the most commonly encountered extrapulmonary TB (EPTB) encountered by participants.

Knowledge regarding the detection of MDR-TB

Eighty eight percent of the participants did a GeneXpert assay before the start of ATT; however only 75.2% of the participants knew that the assay also detects the MTB genome and rifampicin resistance. The awareness of drug sensitivity testing was 87.2% among the participants and 74.4% were aware of the availability of molecular assay platforms for resistance detection to second-line drugs.

Treatment and treatment responses in MDR-TB

Most of the MDR-TB cases were treated in government setting rather than private sector (p-value equals to 0.0093) (Table 4).

**Table 4:** Comparison of knowledge levels of healthcare practitioners in government and private set-ups

Variables		Government Set up (n=164)		Private Set up (n=86)	Odds ratio (95% CI)	p-value
Attended any sensitization programme for Tuberculosis management	Yes	120		59	0.376	0.5399
	No	44		27		
Aware of Index-TB guideline (Indian Guidelines) for extrapulmonary TB	Yes	150	75		0.711	0.3991
	No	14	11			
Follow Index-TB guidelines for the treatment of any Tuberculosis	Always + sometimes	158	76		4.725	0.0297
	Never	6	10			
Encounter of MDR TB in practice	Yes	130	72		0.463	0.4964
	No	34	14			
MDR is a rising problem	Yes	157	80		0.380	0.5376
	No	7	6			
Treat MDR TB patients	Yes	87	30		6.765	0.0093
	No	77	56			
Refer MDR TB patients to MDR-Reference center	Yes	84	42		0.051	0.8222
	No	80	44			
Routine Gene Xpert MTB (CBNAAT/ TruNAAT) before starting ATT	Yes	146	76		0.024	0.8765
	No	18	10			
What does Gene Xpert MTB/Rif assay detect	MTB+ Rifampicin Resistance	125	58		1.791	0.1808
	Any other	39	28			
Awareness regarding Use & availability of Second-line drugs	Yes	107	45		3.427	0.0641
	No	57	41			
Awareness of any molecular assay platform available for resistance detection to Second-line drugs	Yes	118	68		1.150	0.2834
	No	46	18			
Awareness of drug sensitivity tests for AntiTB drugs	Yes	143	75		0.001	0.9975
	No	21	11			
Awareness of use of Linezolid for the treatment of MDR TB	Yes	115	50		3.095	0.0785
	No	49	36			

The decision to complete antitubercular therapy in extrapulmonary TB was made after clinical and radiological improvement among 44% of survey participants, 47.6% also did a bacteriological confirmation to stop ATT, whereas 8.4% of HCPs believed that clinical improvement alone was sufficient to stop anti TB drugs in EPTB.

Multidrug resistance was suspected by HCPs mostly after there was no clinical improvement following 3-6 weeks of trial of ATT. About half of the respondents worked in places that were MDR-TB treatment centers; and 46.8% of the participants were treating MDR-TB patients. More than 85% of the participants believed that patients showed an appropriate response to following the index-TB guidelines (ITBG) (22.4% experienced full recovery of their patients while 64.4% patients had a 75% recovery rate) (Table 2).

## Discussion

This survey provides information regarding the depth of knowledge of MDR-TB among healthcare practitioners and certain lacunae was identified. The survey covered a number of MDR-TB-related topics including pulmonary and extrapulmonary DR-TB as well as sensitization and awareness regarding MDR-TB detection and treatment.

Several knowledge gaps regarding MDR-TB among the doctors were identified. A large number of practitioners (80.8%) admit to having encountered MDR-TB in their clinical practice and the majority (95.2%) of the health care practitioners (HCPs) believe MDR-TB is a rising problem. Another study also reported that antibacterial resistance in the treatment of TB is an important and increasing public health problem [9].

Around 70% participants in our study have attended a TB sensitization program during the last five years; however, awareness regarding the Index TB guidelines stands at around 90%; and is followed significantly more (p-value: 0.0297) in the government setting (Table 4). The MDR-TB patients were more frequently treated in government settings as shown in Table 3. This signifies that the HCPs were acquainted, but training programs need to be imparted more often so as to update them regarding the proper application of these guidelines. The training programs regularly need to emphasize the application of the guidelines into clinical and field practice rather than specifying and discussing the guidelines only. With the nation’s mission of TB eradication by 2025, the pace of the sensitization program needs to be improved. and the guidelines need to be more strictly followed [2].

The duration of treatment is specifically defined for various forms of EPTB in the ITBG [7,8]. TB lymphadenitis is the most common form of EPTB followed by bone TB and pleural TB has been encountered by the clinicians who have participated in this study. Bone TB might be seen as a more common occurrence as a significant number of orthopedics specialists have participated in this study. But roughly one-third of the HCPs still did not follow the ITBG for treatment of EPTB patients; which has shown 100% favorable results for only about 22.4 % of health care professionals; 64.4% of HCPs had favorable outcomes in 75% of cases. Suleiman et al. conducted a study in north-western Somalia also regarding the knowledge about modern TB management among doctors. The authors report that two-thirds of the doctors knew the major symptoms and the important diagnostic procedures, but only a few indicated a correct treatment regimen and followed the guidelines properly [10]. The government of India has made TB a notifiable disease with incentives and rewards for both HCPs and patients [7]. However, some HCPs did not follow ITBG because they were following institutional guidelines. There should be periodic awareness programs regarding MDR-TB, as inadequate and inappropriate treatment is one of the preventable causes of disease relapse and drug resistance.

A study conducted in Iran found 50% of clinicians have knowledge of and adhere to correct treatment guidelines [11]. The results of this study indicate a lack of knowledge, which poses a threat to TB control in the country and can be the reason for emerging as well as spreading drug resistance. In another study conducted in Peru; only 27% of HCPs correctly assessed treatment outcomes [4]. Both clinical and radiological improvements are imperative to continue the course of ATT and to stop after completion. The bacteriological confirmation may not always be feasible at the beginning or completion of treatment especially for EPTB [7]. Attempts to confirm the diagnosis should be accelerated if there is provision for the utilization of a molecular platform like the GeneXpert assay in the initial phase. Unaltered clinical conditions or even worsening of the scenario 3-6 weeks after the trial of standard therapy in cases of proven TB, could be due to MDR-TB. Cartridge based nucleic acid amplification tests (CBNAAT) or the GeneXpert assay and TRUNAT (a chip based micro-PCR platform) assays are really helpful for the initial diagnosis of Rifampicin resistance, can also act as a surrogate marker for MDR TB. These tests are rapid, simpler and have high sensitivity and specificity [12]. In our survey, 88.8% of practitioners use GeneXpert for initial diagnosis. Though, the ITBG has not mandated this test and even a biopsy is not made mandatory and strong clinical suspicion fulfills the requirement to start ATT, this molecular platform should be utilized for the early and prompt management of MDR-TB. More centers including private settings should be equipped with such testing facilities to add in the early diagnosis of MDR-TB. One study from South Africa revealed that clinicians did not adhere to the diagnostic guidelines of the national program. An astounding 85% of PTB cases were diagnosed in absence of sputum microscopy. Chest x-rays alone were used to diagnose PTB in 45% of the records reviewed. In addition, clinicians failed to document a clinical history suggestive of TB. They also showed that only 66 (29%) of the hospital’s 225 smear-positive PTB patients reached the clinics for the completion of their treatment. This shows the dismal state of TB diagnosis and treatment around the globe [13]. A similar study in Southern Mozambique in 2015 also reported the limited use of the GeneXpert assay suggesting that the roll out of the Xpert assay might not have been accompanied by proper education of its applications and its advantages for clinicians and patients. While this technology has been lauded as “a game-changer for TB diagnostics”, a lack of widespread understanding regarding its potential among HCPs and the critical operational requirement of trained laboratory and clinical staff, GeneXpert assay may not be fully adopted [14].

The respondents in our survey largely do not themselves treat MDR-TB. Patients are either referred to an MDR-TB center or a pulmonary medicine specialist. This is encouraging as MDR-TB needs more close monitoring in specialized MDR-TB designated centers with specialists to treat it. Linezolid is an antibiotic that is empirically used to treat MRSA (Methicillin resistant Staphylococcus aureus) in many parts of the country, and it is also a second line anti-tubercular drug. Only 66% of the current study respondents knew about the use of linezolid in the treatment of MDR-TB which again unmasks the gravity of the lack of updates of HCPs regarding treatment options [1,2,15]. These knowledge gaps regarding MDR-TB were also identified by other studies. In one study, the authors reported that 59.2% of their study participants strongly agreed that the indiscriminate use of anti-TB drugs could be a major factor attributing to MDR-TB [9]. This could be due to the fact that most of the sensitization and training programs in our country are held in the government settings, but in a high burden country like India, the training programs should be robust and rigorous and should be conducted in every setting (both private and government) on a regular basis to achieve the ultimate goal of TB elimination by 2025 [3].

Health care practitioners believe that MDR-TB is an emerging issue that can hit us hard in the coming years. If MDR-TB cases are not diagnosed early and treated appropriately, their spread can cause medical havoc. This could be a good starting point for organizations to educate and conduct training programs as one of the approaches to reducing the MDR-TB burden in high incidence countries like ours.

There are certain limitations to this study. Firstly, the sample size of 250 HCPs who consented to the study may be small in regards to the enormous number of HCPs in our country. The respondents may be clustered and not be a true pan India representation. Only allopathic HCPs were included but in many rural places AYUSH (Ayurveda, Yoga and Naturopathy, Unani, Siddha and Homeopathy) doctors also practice and treat TB. The implementation of NTEP largely also depends on stakeholders in the delivery of ATT. Singh et al. had found that Accredited Social Health Activist (ASHA) workers also revealed inadequate knowledge and unsatisfactory practice regarding the then ongoing directly observed therapy (DOT) provision among ASHA workers. The HCPS is one arm, the other arm is the ASHA workers. This signifies the improvement of knowledge at the grass-roots level [16].

Disease burden can be lowered in any community by well-informed HCPs who can educate patients, as well as the general population about TB, increasing resistance, and emerging MDR-TB isolates. With the current level of knowledge about MDR-TB case management, there is clearly a need for the training of doctors in both the public and private sectors. While most sensitization programs in our country are for pulmonary and extrapulmonary TB, there must be programs for MDR-TB.

## Conclusions

The greatest effort in a long run lies in the last lap. India is among the high burden countries for TB is now heading towards TB elimination by 2025. The pace of sensitization programs needs to be accelerated at each level of the pyramid, starting from the grass-roots level of field workers to the apex level of policymakers. With the provision of CBNAAT assays at peripheral centers in addition to district, intermediate and national reference laboratories, there should be improved awareness among HCPs regarding its advantages; otherwise, the provisions shall remain underutilized in the wake of rising incidence as well as increasing drug resistance. NTEP guidelines can be further updated and revised so as to incorporate the GeneXpert assay as a mandatory test before the start of anti-tubercular therapy.
